# The Dark Triad Traits and the Prediction of Eudaimonic Wellbeing

**DOI:** 10.3389/fpsyg.2021.693778

**Published:** 2021-11-05

**Authors:** Yaqian Liu, Na Zhao, Min Ma

**Affiliations:** School of Sociology and Psychology, Central University of Finance and Economics, Beijing, China

**Keywords:** Machiavellianism, narcissism, psychopathy, family support, hedonic well-being, eudaimonic well-being, the Dark Triad Traits

## Abstract

Although numerous studies have focused on personality traits related to well-being, the relationship between the Dark Triad Traits and eudaimonic well-being is still unclear. The purpose of the present study was to explore how the Dark Triad Traits (i.e., narcissism, Machiavellianism, and psychopathy) affect eudaimonic well-being. Further, this study also aimed to explore the mediation effect of family support and hedonic wellbeing. The results showed that the present model had a good model fit (χ^2^/df = 1.91, *p* < 0.001, comparative-fit-index (CFI) = 0.96, tucker-lewis-index (TLI) = 0.95, root mean square error of approximation (RMSEA) = 0.04, standardized root mean square residual (SRMR) = 0.04). There is a significant association between the Dark Triad Traits and eudaimonic wellbeing. Specifically, narcissism directly predicts eudaimonic wellbeing, while the effects of Machiavellianism and psychopathy on eudaimonic wellbeing are serial two-mediator models, which are mediated by family support and hedonic wellbeing. The results would enrich theoretical studies on personality while providing some practical evidence on how to improve the subjective well-being of individuals.

## Introduction

Numerous studies have paid attention to the association between personality traits and wellbeing (e.g., Henningsgaard and Arnau, 2008; Lavigne et al., 2013; Burton et al., 2015). The relationship between the Dark Triad Traits (i.e., Machiavellianism, psychopathy, and narcissism) and wellbeing has also been well-documented (Volmer et al., 2016; Joshanloo, 2021; Van Groningen et al., 2021). Most of these studies had focused on hedonic wellbeing, a kind of wellbeing, which emphasized the momentary emotional balance (Paleari et al., 2021). It is important to indicate that well-being is a complex construct that refers to both optimal psychological experience and functioning (Ryan and Deci, 2001). Different from hedonic wellbeing, eudaimonic wellbeing reflects the “true self,” which focuses on the subjective cognitive-affective experience, such as the experience of meaning and purpose in life (Paleari et al., 2021).

The current study intended to investigate the relationship between the Dark Triad Traits and eudaimonic wellbeing. The study of the issue would extend our understanding of the Dark Triad Traits in two ways: first, investigating the Dark Triad Traits of people in collectivistic culture can extend the understanding of its cultural universality, while the Dark Triad Traits were originated from Western culture, and most previous studies had relied on Western samples. Second, the study would explore the psychological mechanisms between the Dark Triad Traits and eudaimonic wellbeing, which can provide some practical implications in improving the subjective well-being of individuals.

### The Dark Triad Traits and Eudaimonic Wellbeing

The Dark Triad Traits include Machiavellianism, psychopathy, and narcissism (Paulhus and Williams, 2002). Machiavellianism is characterized by a tendency to manipulate others in a cold way to achieve their own ends (Jakobwitz and Egan, 2006). Those who score high on the psychopathy scale tend to seek excitement (Rauthmann and Will, 2011), whereas narcissists tend to show dominance and egoism and usually experience a feeling of superiority over others (Lee and Ashton, 2005). In general, most studies have demonstrated that these dark traits are highly associated with negative psychological outcomes, such as disagreeableness, emotional coldness, aggressiveness, and lack of empathy among others (Miao et al., 2018). Additionally, these dark traits also have many negative effects on the behaviors of individuals, resulting in deception, aggression, impulsive behavior, bullying, and cigarette, alcohol, and substance abuse (Jones and Paulhus, 2010).

Eudaimonic wellbeing is one of subjective wellbeing, which consists of meaningful activity, actualizing one’s potential, and understanding the meaning of life rather than hedonic experiences (Joshanloo, 2021). Previous research has suggested that interpersonal relationship contributes greatly to meaning in the life of individuals (Zhao et al., 2017). However, characterized by coldness and disagreeableness, Machiavellianism and psychopathy are maladaptive especially in the interpersonal domain (Paulhus and Williams, 2002; Jonason et al., 2015). Accordingly, many studies have found that Machiavellianism and psychopathy predict eudaimonic wellbeing negatively; those who score high on these two dimensions would experience less eudaimonic wellbeing (Bartels and Pizarro, 2011; Egan et al., 2014; Aghababaei and Błachnio, 2015). However, when it comes to narcissism, things become more complicated (Zondag, 2005; Ng et al., 2014; Abeyta et al., 2017). Some studies have shown that narcissism has a negative effect on eudaimonic wellbeing (e.g., Ng et al., 2014); while some other studies have found that narcissism is a positive predictor of eudaimonic wellbeing (e.g., Abeyta et al., 2017). We argued that narcissism, as the “brighter” side of the dark traits, would have a positive association with eudaimonic wellbeing (Zondag, 2005; Aghababaei and Błachnio, 2015). Many studies have indicated that narcissists are good at facilitating the active and passive accruals of a social network (Jonason and Schmitt, 2012). They tend to make a better impression in the initial encounter with others and thus were more popular, and they could also be attractive leaders who have a lot of followers (Furtner et al., 2011; Rauthmann, 2012). Moreover, narcissists think highly of judgments and evaluations of others, especially in the context of Chinese culture, which was characterized by collectivism (Raskin and Terry, 1988; Cai et al., 2012). Therefore, we proposed the first hypothesis:

Hypothesis 1: *Machiavellianism and psychopathy would negatively predict eudaimonic wellbeing, while narcissism would positively predict it.*

### The Mediation Effect of Family Support

Researchers have found that social support from significant others contributes greatly to meaning in the life of one (Dunn and O’Brien, 2009; Lee et al., 2017). The results of a longitudinal study also show that early social support significantly predicts a later sense of meaning in life (Krause, 2007). Familialism is considered particularly important in collectivistic cultures, and support from family members can be an extremely important source of social support for people in collectivistic cultures (Chen et al., 2007; Cai et al., 2012). Consistent with the above views, many studies have found that family support can greatly improve the eudaimonic well-being of Chinese people, among both children and adults (Jiang et al., 2016). Therefore, we proposed that social support from the family would be a positive predictor of eudaimonic wellbeing (Zhao and Zhao, 2020).

However, individuals with different personality traits have different perceptions of family support (Nakayama, 2010; Láng and Birkás, 2014). Studies have argued that high emotional intelligence individuals could perceive more social support and have better social relationships (Salovey et al., 2002). In contrast, those who score high on dark personality traits (i.e., Machiavellianism and psychopathy) are emotionally deficit (Jonason and Krause, 2013). Therefore, individuals with high Machiavellianism and high psychopathy are not likely to feel social support from their family members (Láng and Birkás, 2014). Meanwhile, narcissists, who tend to seek outside attention and recognition and have higher emotional intelligence, emphasize the importance of family support and thus perceive more of it (Nakayama, 2010; Jonason and Schmitt, 2012). Based on this understanding, we proposed the second hypothesis:

Hypothesis 2: *The link between the Dark Triad Traits and eudaimonic wellbeing is mediated by family support.*

### The Mediation Effect of Hedonic Wellbeing

Hedonic wellbeing and eudaimonic wellbeing are two concepts that are closely related (Fowersa et al., 2010). Different from eudaimonic wellbeing focusing on the meaning in life, hedonic wellbeing refers to the general satisfaction with present life (Joshanloo, 2019). As an important source of meaning, hedonic wellbeing contributes greatly to eudaimonic wellbeing (Howell et al., 2013). Specifically, those with a higher level of hedonic wellbeing usually experience greater eudaimonic well-being; hedonic wellbeing could be a strong predictor of eudaimonic wellbeing (Gao et al., 2014; Zhao and Zhao, 2020). However, Machiavellianism and psychopathy are associated with lower positive moods, and they have a negative effect on hedonic wellbeing (Egan et al., 2014; Kaufman et al., 2019). Meanwhile, narcissism is associated with a more positive mood and can be a positive predictor of hedonic wellbeing (Egan et al., 2014; Van Groningen et al., 2021). Therefore, the third hypothesis was proposed as follows:

Hypothesis 3: *Hedonic well-being would play a mediating role in the relationship between the Dark Triad Traits and eudaimonic well-being*.

### The Relationship Between Family Support and Hedonic Well-Being

As an important buffer for stress and other negative life events, social support from family could improve the life quality of individuals and could be a significant source of hedonic wellbeing (Chu et al., 2010). Studies have also shown that perceived family support positively predicts the hedonic well-being of individuals (Heng et al., 2020; Ling et al., 2020).

Combined with the above arguments, we argued that individuals with high Machiavellianism and psychopathy would be less likely to feel support from family, thus experiencing less hedonic wellbeing and less eudaimonic wellbeing. Meanwhile, narcissists seemed to perceive more family support and thereby would have a higher level of hedonic and eudaimonic wellbeing. Therefore, we proposed a chain mediation model as our fourth hypothesis:

Hypothesis 4: *The Dark Triad Traits would indirectly predict the eudaimonic well-being of individuals through the intermediary chain from family support to hedonic wellbeing.*

In addition, many studies have indicated that demographic variables, such as gender, age, marital status, and educational level, could affect the eudaimonic well-being of individuals (Zhao and Xue, 2019; Fu et al., 2021; Lu et al., 2021), thus these demographic variables were tested in the present study.

## Materials and Methods

### Participants

This study was approved by the Ethics Committee of the Department of Psychology of the Central University of Finance and Economics. The survey was carried out through Sojump (a survey company) in China in 2020. Through online advertisements and the WeChat Moments, by convenient sampling, samples of 737 adults were recruited. All participants completed a questionnaire booklet that included (1) the Dark Triad Traits, (2) eudaimonic wellbeing, (3) hedonic wellbeing, and (4) family support. Each question in the questionnaire was compulsory, and the participants could not submit the link without answering all the questions. All subjects participated voluntarily after providing informed consent, and no incentive was provided to them. Thirty-two participants were excluded since their time to fill in the questionnaire was less than 300 s, with a response rate of 95.66%. The samples of 705 adults (278 males, 39.4%; *M*_age_ = 25.46 years, *SD*_age_ = 7.98 years) covered Wuhan, Zhejiang, Bejing, He’nan, Fujian provinces. As for their education and civil status, 6.8% of the participants had attained an educational level of senior high school or below, 93.2% had a bachelor’s degree or above; 25.2% were married, 74.2% were unmarried, 0.1% were widowed, and the rest 0.5% were divorced or separated.

### Measurements

#### The Dark Triad Traits

The validated Chinese version of the *Dirty Dozen* scale (Jonason and Webster, 2010) was used in the current study (Geng et al., 2015). The scale consists of three subscales: Machiavellianism (e.g., “I tend to manipulate others to get my way”), psychopathy (e.g., “I tend to not be too concerned with morality or the morality of my actions”), and narcissism (e.g., “I tend to expect special favors from others”). Each subscale consists of four items with a total of 12 items. Participants were asked to rate each item on a 7-point scale from 1 (“definitely does not apply”) to 7 (“definitely applies.”) Higher total scores for all items indicated greater levels of dark personality traits. Cronbach’s alphas for the three dimensions (Machiavellianism, psychopathy, and narcissism) were 0.88, 0.75, and 0.86, respectively. Three latent variables were created for the Dark Triad Traits. The residuals of items 9 and 10 in the narcissistic dimension were correlated. The fit of the measurement model of the Dark Triad Traits was acceptable, χ^2^ (50) = 205.33, *p* < 0.001, comparative-fit-index (CFI) = 0.97, tucker-lewis-index (TLI) = 0.95, Root Mean Square Error of Approximation (RMSEA) = 0.07, standardized root mean square residual (SRMR) = 0.04, with factor loadings ranging from 0.56 to 0.90.

#### Eudaimonic Well-Being

Participants completed the 10-item Meaning in Life Questionnaire (MLQ), which represented eudaimonic wellbeing in a previous study (Nelson et al., 2014). The item named “My life has no clear purpose” was deleted because of its too low factor loading. Therefore, the final version of the scale consists of nine items. Sample items include “I understand my life’s meaning” and “I am always searching for something that makes my life feel significant.” The participants were asked to indicate the extent to which they agreed or disagreed with each statement on a 7-point scale (1 = “completely disagree”; 7 = “completely agree”), where higher scores indicate higher levels of eudaimonic wellbeing. The Cronbach’s alpha for the current study was 0.91. A latent variable for eudaimonic wellbeing was created. The residuals of items 2, 3, 7, 8, and 10 were correlated. The fit of the measurement model of eudaimonic wellbeing was acceptable, χ^2^ (19) = 109.69, *p* < 0.001, CFI = 0.98, TLI = 0.96, RMSEA = 0.08, SRMR = 0.05, with factor loadings ranging from 0.37 to 0.88.

#### Hedonic Well-Being

Life satisfaction, as a key component of subjective wellbeing, is usually used to assess hedonic wellbeing (Ryan and Deci, 2001). A validated Chinese version of the Satisfaction with Life Scale (SWLS) comprising five items was used to measure hedonic wellbeing. We asked the participants to indicate the extent to which they agreed or disagreed with the items of this scale (e.g., “I am satisfied with my life.”) on a 7-point Likert scale (1 = “completely disagree”; 7 = “completely agree”), where higher scores indicate higher levels of hedonic wellbeing. The Cronbach’s alpha for the current study was 0.90. The residuals of items 4 and 5 were correlated. The fit of the measurement model of hedonic wellbeing was acceptable, χ^2^ (4) = 16.95, *p* < 0.01, CFI = 0.99, TLI = 0.99, RMSEA = 0.07, SRMR = 0.01, with factor loadings ranging from 0.69 to 0.90.

#### Family Support

To assess people’s perceived family support, we used 15 items taken from the Perceived Social Support from Friends and from Family Scales (Procidano and Heller, 1983). The scale was translated from English to Chinese. Considering the differences in the expressions of these two languages, we deleted item 4 (named “When I confide in members of my family, it makes me uncomfortable”), item 11 (named “When I confide in the members of my family who are closest to me, I get the idea that it makes them uncomfortable”), and item 12 (named “My family is sensitive to my personal needs”), which had vague semantic meanings and too low factor loadings. Therefore, the final version of the scale consists of 12 items (e.g., “My family gives me the moral support I need”). As for scoring, “Yes” corresponds to 1, and “No” stands for 0. Items 10, 14, and 15 were reversing items. Higher scores indicate a stronger perception of family support. The Cronbach’s alpha for the current study was 0.84. The residuals of items 10, 14, and 15 were correlated. The fit of the measurement model of perceived family support was acceptable, χ^2^ (51) = 156.48, *p* < 0.001, CFI = 0.96, TLI = 0.95, RMSEA = 0.05, SRMR = 0.03, with factor loadings ranging from 0.30 to 0.80.

### Statistical Analyses

Descriptive statistics for all study variables and correlations of key variables were obtained using SPSS 21.0. Structural equation modeling (SEM) was used to test the hypotheses using Mplus 8.3.

## Results

### Descriptive Results

Descriptive statistics and correlations for all variables are presented in Table 1. The results indicated that there was a significant positive correlation among the three dimensions of the Dark Triad Traits. Moreover, the three dark triad personality traits were negatively associated with family support. Psychopathy was negatively associated with hedonic well-being, while both Machiavellianism and psychopathy were negatively associated with eudaimonic well-being. In addition, family support was also positively correlated with hedonic well-being. Family support and hedonic well-being both were positively correlated with eudaimonic wellbeing.

**TABLE 1:** Descriptive statistics and correlations for all study variables (*n* = 705).

**Variable**	***M (SD)***	**1**	**2**	**3**	**4**	**5**	**6**
1. Age	25.46 (7.98)						
2. Machiavellianism	7.17 (4.31)	–0.05					
3. Psychopathy	7.81 (4.36)	–0.05	0.66***				
4. Narcissism	15.13 (5.99)	–0.05	0.36***	0.34***			
5. Family support	8.75 (3.16)	0.16***	−0.16***	−0.29***	−0.14***		
6. Hedonic well-being	20.53 (7.41)	0.12**	0.02	−0.12***	–0.06	0.33***	
7. Eudaimonic well-being	5.05 (1.08)	0.06	−0.13***	−0.20***	0.05	0.29***	0.51***

*All the means, variances, and correlations of the variables were estimated using SPSS 21.0.*

** *p* < 0.01, *** *p* < 0.001.

### Analysis of Demographic Variables

Demographic variables (i.e., gender, age, marriage, and education) were tested in the present study. The results suggested that gender [*t* (703) = 0.79, *p* = 0.429] and age [β = 0.06, *p* = 0.114] had no significant effect on eudaimonic well-being. We handled the marital status as two groups (married/others) and found that there was no significant difference between the two groups on eudaimonic wellbeing, *t* (703) = 1.57, *p* = 0.118. However, educational level had a significant effect on eudaimonic wellbeing, *F* (4, 700) = 3.65, *p* < 0.01. Therefore, only education was controlled for in the SEM.

### Structural Model Analysis

A latent structural analysis was used to explore the relationship among the Dark Triad Traits, family support, hedonic wellbeing, and eudaimonic wellbeing. To formally test the indirect effects, 95% CIs were computed from 1,000 bootstrap samples. In general, when CIs exclude zero, we can conclude that mediation is significant. After controlling for education, a good model fit was attained, χ^2^ (673) = 1,282.22, χ^2^/*df* = 1.91, *p* < 0.001, CFI = 0.96, TLI = 0.95, RMSEA = 0.04, SRMR = 0.04.

**FIGURE 1:**
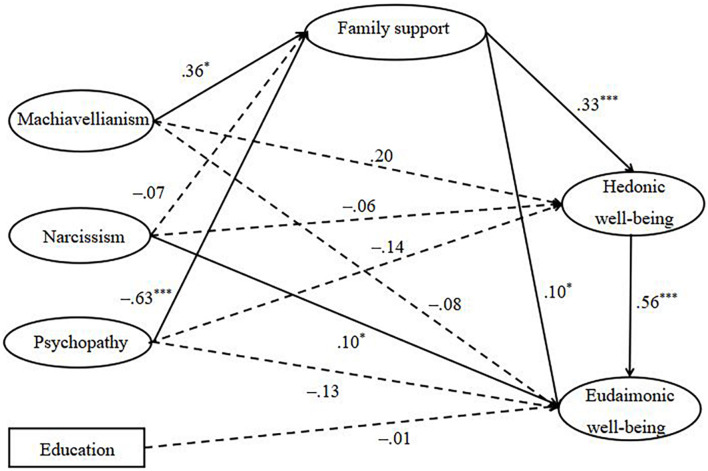
The role of the DarkP Triad Trait on eudaimonic wellbeing of individuals *via* perceived family support and hedonic well-being. Values presented are standardized coefficients. The dash lines represent non-significant paths. Endogenous error correlations and control variables have not been drawn for parsimony. ^∗^ *p* < 0.05, ^∗∗∗^ *p* < 0.001.

All direct paths in our model are shown in Figure 1. Machiavellianism (β = −0.08, *SE* = 0.13, *p* = 0.505) and psychopathy (β = −0.13, *SE* = 0.14, *p* = 0.356) had no significant direct effect on eudaimonic wellbeing, but the direct effect of narcissism on eudaimonic wellbeing was significant (β = 0.10, *SE* = 0.04, *p* < 0.05). The results also illustrated that psychopathy negatively predicted family support (β = −0.63, *SE* = 0.16, *p* < 0.001), Machiavellianism positively predicted it (β = 0.36, *SE* = 0.15, *p* < 0.05), while narcissism had no significant effect on it (β = −0.07, *SE* = 0.04, *p* = 0.070). Machiavellianism (β = 0.20, *SE* = 0.11, *p* = 0.069), narcissism (β = −0.06, *SE* = 0.04, *p* = 0.115), and psychopathy (β = −0.14, *SE* = 0.12, *p* = 0.246) all had no significant effect on hedonic wellbeing. Family support positively predicted hedonic wellbeing (β = 0.33, *SE* = 0.05, *p* < 0.001) and eudaimonic well-being (β = 0.10, *SE* = 0.05, *p* < 0.05). Hedonic wellbeing had a significant positive effect on eudaimonic wellbeing (β = 0.56, *SE* = 0.04, *p* < 0.001). Education had no significant effect on eudaimonic wellbeing (β = −0.01, *SE* = 0.04, *p* = 0.808).

To further examine the indirect effects, we conducted bootstrapping analyses based on 1,000 bootstrap resamples. The results showed that the total indirect effect of narcissism on eudaimonic wellbeing was significant, β = −0.05, *SE* = 0.03, *p* < 0.05, 95% *CI* = (−0.11, −0.002). However, the indirect effect of narcissism on eudaimonic wellbeing through perceived family support was not significant [β = −0.01, *SE* = 0.01, *p* = 0.223, 95% *CI* = (−0.02, 0.001)], and neither was it mediated by hedonic wellbeing [β = −0.03, *SE* = 0.02, *p* = 0.114, 95% *CI* = (−0.08, 0.01)] nor the intermediary chain from perceived family support to hedonic wellbeing [β = −0.01, *SE* = 0.01, *p* = 0.085, 95% *CI* = (−0.03, 0.001)].

The total indirect effect of Machiavellianism on eudaimonic wellbeing was significant [β = 0.22, *SE* = 0.08, *p* < 0.01, 95% *CI* = (0.09, 0.41)]. Specifically, the indirect effect of Machiavellianism on eudaimonic wellbeing through the intermediary chain from perceived family support to hedonic wellbeing was significant [β = 0.07, *SE* = 0.03, *p* < 0.05, 95% *CI* = (0.02, 0.14)], but the indirect effects through perceived family support [β = 0.04, *SE* = 0.03, *p* = 0.134, 95% *CI* = (−0.001, 0.09)] and through hedonic wellbeing [β = 0.11, *SE* = 0.06, *p* = 0.075, 95% *CI* = (−0.004, 0.25)] were not significant.

The total indirect effect of psychopathy on eudaimonic wellbeing was significant, β = −0.26, *SE* = 0.09, *p* < 0.01, 95% *CI* = (−0.47, −0.13). Specifically, the indirect effect of psychopathy on eudaimonic wellbeing through the intermediary chain from perceived family support to hedonic wellbeing was significant [β = −0.12, *SE* = 0.03, *p* < 0.01, 95% *CI* = (−0.19, −0.06)], but the indirect effects through perceived family support [β = −0.06, *SE* = 0.04, *p* = 0.078, 95% *CI* = (−0.14, 0.002)] and through hedonic wellbeing [β = −0.08, *SE* = 0.07, *p* = 0.251, 95% *CI* = (−0.23, 0.05)] were not significant.

## Discussion and Conclusion

The purpose of the current study is to examine the predictive role of the Dark Triad Traits on eudaimonic wellbeing, the relationship of which is mediated by family support and hedonic wellbeing. Findings demonstrated that narcissism predicted eudaimonic wellbeing directly and positively, while Machiavellianism and psychopathy predicted eudaimonic well-being as mediated by family support and hedonic wellbeing.

In line with most prior research (Zondag, 2005; Rauthmann and Kolar, 2012; Aghababaei and Błachnio, 2015), our findings indicate the “brighter” side of the narcissism trait. That is, narcissists are not only more successful at work and have better social relationships, but they also intend to experience more eudaimonic wellbeing (Rauthmann, 2012; Abeyta et al., 2017). However, the present study did not distinguish different types of narcissism. Rose (2002) has proposed covert and overt narcissism as two subtypes of narcissism. Overt narcissists tend to rely on overt strategies to regulate the self (e.g., self-enhancement and devaluing others), while covert narcissists are more likely to seek social approval to modulate their fragile egos (Ng et al., 2014). Therefore, the experience of subjective wellbeing may vary depending on the types of narcissism (Zondag et al., 2009). Future research should distinguish between the two subtypes and further investigate the role of covert and overt narcissism in predicting the subjective well-being of individuals.

Our results suggest that family support plays an important role in the relationship between the Dark Triad Traits and wellbeing. The findings extend our understanding of the importance of family support to Chinese people. Previous studies have demonstrated that a sense of home or family may be essential in collectivistic cultures (Yang et al., 2020). China is a classical collectivistic country and Chinese people often report higher familial (rather than dyadic connections or the individual) than some individualistic countries (Cai et al., 2012; Chang et al., 2016). In the present study, for those who scored higher in Machiavellianism and psychopathy, family support still played an indispensable role in subjective wellbeing (both hedonic and eudaimonic wellbeing). The results also enlighten us that family members can provide people with more social support to enhance their subjective well-being.

Interestingly, we have found a positive effect of Machiavellianism, which is contrary to our hypothesis. Many previous studies on the Dark Triad Traits have indicated that Machiavellianism is as “dark” as psychopathy, and it has repeatedly been proven to have a significant adverse impact on physical and mental health (Egan et al., 2014; Aghababaei and Błachnio, 2015; Zhu et al., 2021). However, our results might imply a powerful adaptive function of Machiavellianism in Chinese society. We propose two potential explanations for the inconsistency: first, Machiavellianism originated and developed in the Western value system, and literature on it relied mostly on samples from Western countries (Rogoza et al., 2020); thus, more studies in collectivistic culture are needed to further examine whether cross-cultural differences exist. Second, researchers have found that as societies become more advanced, citizens would become more Machiavellian (Jonason et al., 2020). In the rapidly developing Chinese society, utilitarianism and Machiavellianism have become more popular (Zhao and Liao, 2013). Therefore, with more people becoming Machiavellian, Machiavellians may receive and experience more social support from significant others and thus experience higher subjective well-being. Further research is needed to explore this interesting finding.

However, there are some limitations of the present study. First, this study was based on cross-sectional data, so the causal relationships could not be exactly determined from the current study; therefore, future studies need a longitudinal design to interpret cause and effect and direction of causality. Second, the present study relied on questionnaires from a single source (i.e., self-reports of individuals) to collect data. These self-report measures are vulnerable to response bias (e.g., recall bias and social desirability), and the effects may be overestimated due to shared method variance. It is important to consider the extent to which such self-reports converge with other measures, such as informant reports or observations of actual behavior. Third, the description of the characteristics of Machiavellianism originated from the West, which was very straightforward (e.g., “I tend to manipulate others to get my way”). Nevertheless, Chinese people generally prefer more reserved expressions, thus they may tend to choose smaller points when filling in the scale. According to the results of the present study, with the mean of 7.17 (the total scores of four items), the scores of Machiavellianism were much lower than that of psychopathy and narcissism, thus we might be experiencing the floor effect. Therefore, appropriate caution should be used in interpreting the above findings.

## Data Availability Statement

The original contributions presented in the study are included in the article/Supplementary Material, further inquiries can be directed to the corresponding author.

## Ethics Statement

The studies involving human participants were reviewed and approved by Psychological Ethics Committee of Central University of Finance and Economics. The patients/participants provided their written informed consent to participate in this study.

## Author Contributions

All authors listed have made a substantial, direct and intellectual contribution to the work, and approved it for publication.

## Conflict of Interest

The authors declare that the research was conducted in the absence of any commercial or financial relationships that could be construed as a potential conflict of interest.

## Publisher’s Note

All claims expressed in this article are solely those of the authors and do not necessarily represent those of their affiliated organizations, or those of the publisher, the editors and the reviewers. Any product that may be evaluated in this article, or claim that may be made by its manufacturer, is not guaranteed or endorsed by the publisher.
