# Supplementary material for: The Dark Triad Traits and the Prediction of Eudaimonic Wellbeing
Source: Front Psychol. 2021 Nov 5;12:693778. doi: 10.3389/fpsyg.2021.693778 (PMC8602073; doi:10.3389/fpsyg.2021.693778)
Supplement: Supplementary file 1 [file Data_Sheet_1.ZIP › questionnaire.docx]

**一、请标明你在多大程度上同意下列陈述。**

|  |  | 非常  不同意 |  |  |  |  |  | 非常  同意 |
| --- | --- | --- | --- | --- | --- | --- | --- | --- |
| 1 | 在大多数情况下我的生活是接近我的理想的。 | 1 | 2 | 3 | 4 | 5 | 6 | 7 |
| 2 | 我的生活条件非常好。 | 1 | 2 | 3 | 4 | 5 | 6 | 7 |
| 3 | 我对我的生活感到满意。 | 1 | 2 | 3 | 4 | 5 | 6 | 7 |
| 4 | 目前为止我已经得到了生活中我想得到的重要东西。 | 1 | 2 | 3 | 4 | 5 | 6 | 7 |
| 5 | 如果可以再活一次，我还愿意过现在这样的生活。 | 1 | 2 | 3 | 4 | 5 | 6 | 7 |

**二、首先，请您花一点时间思考一下，“对您来说，什么使你感觉到您的生活是很重要的”。然后，根据下列的描述与您的情况相符合的程度，在1-7中做出选择。**

|  |  | 非常  不同意 |  |  |  |  |  | 非常  同意 |
| --- | --- | --- | --- | --- | --- | --- | --- | --- |
| 1 | 我很了解自己的人生意义。 | 1 | 2 | 3 | 4 | 5 | 6 | 7 |
| 2 | 我正在寻找某种使我的生活有意义的东西。 | 1 | 2 | 3 | 4 | 5 | 6 | 7 |
| 3 | 我总是在寻找自己人生的目标。 | 1 | 2 | 3 | 4 | 5 | 6 | 7 |
| 4 | 我的生活有很明确的目标感。 | 1 | 2 | 3 | 4 | 5 | 6 | 7 |
| 5 | 我很清楚是什么使我的人生变得有意义。 | 1 | 2 | 3 | 4 | 5 | 6 | 7 |
| 6 | 我已经发现了一个令人满意的人生目标。 | 1 | 2 | 3 | 4 | 5 | 6 | 7 |
| 7 | 我一直在寻找能使我的生活感觉起来是重要的东西。 | 1 | 2 | 3 | 4 | 5 | 6 | 7 |
| 8 | 我正在寻找自己人生的目标和“使命”。 | 1 | 2 | 3 | 4 | 5 | 6 | 7 |
| 9 | 我的生活没有很明确的目标。 | 1 | 2 | 3 | 4 | 5 | 6 | 7 |
| 10 | 我正在寻找自己人生的意义。 | 1 | 2 | 3 | 4 | 5 | 6 | 7 |

**三、请标明你在多大程度上同意下列陈述。**

|  |  | 1完全不符合 | 2  比较不符合 | 3  有点不符合 | 4  不确定 | 5  有点符合 | 6  比较符合 | 7  完全符合 |
| --- | --- | --- | --- | --- | --- | --- | --- | --- |
| 1 | 我倾向于操纵别人以达到自己的目的。 | 1 | 2 | 3 | 4 | 5 | 6 | 7 |
| 2 | 我习惯于欺骗别人以达到自己的目的 | 1 | 2 | 3 | 4 | 5 | 6 | 7 |
| 3 | 我习惯于奉承别人达到自己的目的。 | 1 | 2 | 3 | 4 | 5 | 6 | 7 |
| 4 | 我倾向于利用别人达到自己的目的。 | 1 | 2 | 3 | 4 | 5 | 6 | 7 |
| 5 | 我缺乏悔恨之心。 | 1 | 2 | 3 | 4 | 5 | 6 | 7 |
| 6 | 我不太关心自己的行为是否符合道德规范。 | 1 | 2 | 3 | 4 | 5 | 6 | 7 |
| 7 | 我冷酷、麻木。 | 1 | 2 | 3 | 4 | 5 | 6 | 7 |
| 8 | 我愤世嫉俗。 | 1 | 2 | 3 | 4 | 5 | 6 | 7 |
| 9 | 我希望别人赞美我。 | 1 | 2 | 3 | 4 | 5 | 6 | 7 |
| 10 | 我希望别人关注我。 | 1 | 2 | 3 | 4 | 5 | 6 | 7 |
| 11 | 我追求名誉地位。 | 1 | 2 | 3 | 4 | 5 | 6 | 7 |
| 12 | 我期望从别人那里获得特殊礼遇。 | 1 | 2 | 3 | 4 | 5 | 6 | 7 |

**三、请标明你在多大程度上同意下列陈述。**

|  |  | 1代表 “是“ | 2代表“否” |
| --- | --- | --- | --- |
| 1 | 我的家庭给予我所需要的精神上的支持。 | 1 | 2 |
| 2 | 我在感情上依赖家庭的支持。 | 1 | 2 |
| 3 | 我的家庭成员善于帮助我解决问题。 | 1 | 2 |
| 4 | 当我依赖我的家庭成员时，我会感到不舒服。 | 1 | 2 |
| 5 | 我能从我的家庭里得到有关如何去做一些事情的好主意。 | 1 | 2 |
| 6 | 我的家庭愿意听我在想什么。 | 1 | 2 |
| 7 | 我和我家庭中的其他成员能坦诚交谈我们对事情的看法。 | 1 | 2 |
| 8 | 如果我情绪不好，有一个家庭成员可以帮助我。 | 1 | 2 |
| 9 | 我的家庭成员分享我很多感兴趣的事。 | 1 | 2 |
| 10 | 我与家庭成员之间的关系不如别人与他们家庭成员的关系那么密切。 | 1 | 2 |
| 11 | 当我依赖与我最为密切的家庭成员时，我感到这使他们不舒服。 | 1 | 2 |
| 12 | 我的家庭对我的个人需求敏感。 | 1 | 2 |
| 13 | 我与其中的一个家庭成员趣味相投。 | 1 | 2 |
| 14 | 我希望我的家庭与现在有很大的区别。 | 1 | 2 |
| 15 | 其他大部分人与他们家庭的关系要比我密切。 | 1 | 2 |

1. **最后请您填写以下人口统计学信息：**

**1.** 性别： ①男 ②女  **2.** 出生年月： 年 月

**3.** 您的学历或文化程度：①小学及以下; ②初中; ③高中或中专; ④大学; ⑤研究生

**4.** 您目前的婚姻状况：　 ①未婚;　②丧偶;　③离异或分居; ④已婚
